# Cognitive health among older adults in the United States and in England

**DOI:** 10.1186/1471-2318-9-23

**Published:** 2009-06-25

**Authors:** Kenneth M Langa, David J Llewellyn, Iain A Lang, David R Weir, Robert B Wallace, Mohammed U Kabeto, Felicia A Huppert

**Affiliations:** 1 Department of Internal Medicine, University of Michigan, Ann Arbor, MI, USA; 2 Department of Public Health and Primary Care, University of Cambridge, Cambridge, UK; 3 Epidemiology and Public Health, Peninsula Medical School, Exeter, UK; 4 Institute for Social Research, University of Michigan, Ann Arbor, MI, USA; 5 Department of Epidemiology, University of Iowa, Iowa City, IA, USA; 6 Department of Psychiatry, University of Cambridge, Cambridge, UK

## Abstract

**Background:**

Cognitive function is a key determinant of independence and quality of life among older adults. Compared to adults in England, US adults have a greater prevalence of cardiovascular risk factors and disease that may lead to poorer cognitive function. We compared cognitive performance of older adults in the US and England, and sought to identify sociodemographic and medical factors associated with differences in cognitive function between the two countries.

**Methods:**

Data were from the 2002 waves of the US Health and Retirement Study (HRS) (n = 8,299) and the English Longitudinal Study of Ageing (ELSA) (n = 5,276), nationally representative population-based studies designed to facilitate direct comparisons of health, wealth, and well-being. There were differences in the administration of the HRS and ELSA surveys, including use of both telephone and in-person administration of the HRS compared to only in-person administration of the ELSA, and a significantly higher response rate for the HRS (87% for the HRS vs. 67% for the ELSA). In each country, we assessed cognitive performance in non-hispanic whites aged 65 and over using the same tests of memory and orientation (0 to 24 point scale).

**Results:**

US adults scored significantly better than English adults on the 24-point cognitive scale (unadjusted mean: 12.8 vs. 11.4, P < .001; age- and sex-adjusted: 13.2 vs. 11.7, P < .001). The US cognitive advantage was apparent even though US adults had a significantly higher prevalence of cardiovascular risk factors and disease. In a series of OLS regression analyses that controlled for a range of sociodemographic and medical factors, higher levels of education and wealth, and lower levels of depressive symptoms, accounted for some of the US cognitive advantage. US adults were also more likely to be taking medications for hypertension, and hypertension treatment was associated with significantly better cognitive function in the US, but not in England (P = .014 for treatment × country interaction).

**Conclusion:**

Despite methodological differences in the administration of the surveys in the two countries, US adults aged ≥ 65 appeared to be cognitively healthier than English adults, even though they had a higher burden of cardiovascular risk factors and disease. Given the growing number of older adults worldwide, future cross-national studies aimed at identifying the medical and social factors that might prevent or delay cognitive decline in older adults would make important and valuable contributions to public health.

## Background

Cognitive function is a key determinant of independence and quality of life among older adults.[1] Recent demographic, medical and social trends in both the United States and England are likely to have had an important impact on the cognitive health of older adults in both countries. For instance, a recent study reported that adults in the United States were much more likely to have hypertension, obesity, diabetes, and heart disease than adults in England, [2] and there is growing evidence that these cardiovascular risks are also risks for cognitive impairment and dementia among older adults. [3-5] Other important developments over the past two decades on both sides of the Atlantic, such as more effective treatments for hypertension and high cholesterol, new medications for the treatment of Alzheimer's disease, lifestyle changes such as use of cigarettes and alcohol, and rising levels of education and wealth may also have had an impact on cognitive health and function among older adults in the United States and England.[6] At the same time, the organization and level of funding of the health care systems in the two countries are strikingly different, with per capita health care expenditures in the United States nearly 2.5 times higher than in the United Kingdom ($5,700 in the US vs. $2,300 in the UK).[7]

International comparisons of cognitive health have been made difficult by the lack of comparable data on cognitive functioning in different countries. We report here the first comparison of cognitive function in the United States and England, using the same cognitive measures administered to nationally representative samples of older adults in both countries. Our research questions were: 1) Are there differences in cognitive function among adults aged 65 and older in the two countries; and 2) If so, are there differences in demographic, socioeconomic or health measures across the two countries that account for the difference in cognitive function?

## Methods

### Data and Study Populations

We used data from the 2002 waves of the Health and Retirement Study (HRS) and the English Longitudinal Study of Ageing (ELSA). The HRS and ELSA are biennial, longitudinal, nationally representative surveys of, respectively, US adults aged 51 and older, and English adults aged 50 and older.[8,9] The two studies were developed collaboratively with significant overlap in survey questions in order to facilitate valid cross-national comparisons of aging-related changes in health, wealth, and well-being. More detail on the studies, including all survey questions, can be found at the HRS[10] and ELSA[11] web sites.

For this study, we included individuals who were aged 65 and older. To maximize the comparability of the US and English samples, we only included respondents of white race since ELSA contains very few (n = 156) non-white individuals. We also excluded individuals represented by a proxy (1,171 [12%] in the HRS and 96 [2%] in the ELSA) since the cognitive tests were not administered to these respondents. The overall response rate among all eligible respondents was 87% for the 2002 HRS and 67% for ELSA. The final study samples included 8,299 individuals from the HRS and 5,276 individuals from the ELSA.

Both the HRS and ELSA studies include population weights that can be used to draw valid inferences for the entire US and English age 65+ populations, respectively. In the HRS, weights are constructed in a two-step process, where the first step develops post-stratified household weights using the initial sampling probabilities for each household, as well as birth year, race/ethnicity, and gender of household members. The second step uses these household weights to then construct post-stratified respondent-level weights which are scaled to yield weight sums corresponding to the number of individuals in the US population as measured by the US Census Bureau's Current Population Survey (CPS) for the month of March in the year of data collection.

ELSA study participants were drawn from the Health Survey for England, which has an equal probability sample design, so weights to account for selection probabilities are not needed. However, weighting is needed to take into account household and individual non-response which was done by analyzing stage and extent of drop-out from the study, and took into account factors including region of residence, age of oldest person, household size, social class, and incidence of longstanding illness. This information was used in logistic regression models to predict non-response probability and the resulting values were inverted for responding households to provide an initial non-response weight. A second round of weighting was used to adjust the initial household non-response weights to ensure the weighted sample matched the English population as assessed by the 2001 National Census. Full details on the development of population weights for the HRS and ELSA studies are available at the study websites[10,11].

The HRS survey was administered by telephone (71% of the sample) and in-person (29% of the sample). The HRS attempts to interview older respondents (age ≥ 80) in-person whenever possible. All ELSA interviews were performed in-person.

### Measurement of Cognitive Function in the HRS and ELSA

Both the HRS and ELSA assessed cognitive function using tests of immediate and delayed recall of 10 common nouns. A list of ten words was presented orally to study participants, who were then asked to recall as many words as possible immediately after the list was read, and then again after an approximately five-minute delay during which they completed other survey questions. The same four randomly assigned lists of 10 nouns were used in both the HRS and ELSA. Orientation to the day, date, month, and year were also assessed in the same way in both the HRS and ELSA. These three tests resulted in a cognitive scale ranging from 0 to 24 possible points (10 points for immediate recall, 10 points for delayed recall, and 4 points for orientation). If a respondent refused to provide an answer for any of the 3 tests, they were assigned a score of "0" for that test. Ninety-five (1%) HRS respondents and 43 (1%) ELSA respondents refused to answer for one or more of the cognitive tests. Detailed information on the cognitive measures used in this analysis, including their derivation, reliability, and validity, is available at the HRS website.[12]

### Measures of Health Conditions, Risk Factors, and Treatments

Participants in the both the HRS and ELSA were asked about the presence of common chronic health conditions using the question, "Has a doctor ever told you that you had...?" For this analysis, we included stroke, diabetes, heart disease, hypertension, lung disease, and cancer. Depressive symptoms experienced in the last week were assessed using an 8-item version of the Center for Epidemiologic Studies Depression (CES-D) scale.[13] The 8-item version of the CES-D scale used in the HRS and ELSA has comparable reliability and validity to the widely used and validated 20-item CES-D Scale.[13,14].

Smoking status (never, former, or current), and alcohol consumption (average number of days/week that alcohol was consumed over the last 3 months [HRS] or 12 months [ELSA]) were assessed similarly in both the HRS and ELSA. Among those who reported hypertension or diabetes, current use of prescription medications to treat these conditions was determined. (Diabetes treatments were categorized as none, oral medications only, and insulin)

The HRS and ELSA determine the presence of limitations in independent function by asking about difficulty with 6 activities of daily living (ADLs; eating, getting in and out of bed, toileting, dressing, bathing, walking across a room) and 5 instrumental activities of daily living (IADLs; preparing meals, grocery shopping, making phone calls, taking medications, managing money).[15]

### Sociodemographic Measures

We included age (65 – 74, 75 – 84, ≥ 85), gender, household net worth (tertiles; 2002 US dollars) and level of education as sociodemographic measures in the analyses. British pounds were converted to 2002 US dollars using the average currency exchange rate for 2002.[16] Measures of education differ in the HRS and ELSA. Following Banks and colleagues, [2] we identified low, middle, and high education categories for each country (0 to 12 years of school, 13 to 15 years, and ≥ 16 years, respectively, in the US; qualified to lower than "Ordinary-level" [O-level], O-level to lower than "Advanced-level" [A-level], and A-level or higher, respectively, in England).

### Analysis Plan

We calculated scores for the 3 individual cognitive tests (immediate recall, delayed recall, and orientation) as well as for the combined 24-point scale. Mean scores for the full sample, and for age-, gender-, and education-stratified samples for the US and England were compared using t-tests.

We then pooled data from the US and England and estimated an ordinary least squares (OLS) regression model with total cognitive score as the dependent variable. A dichotomous variable indicating the country (0 for England; 1 for the US) was used to determine cross-national differences in cognitive function, after accounting for sociodemographic and health variables. We estimated 8 separate linear regression models with different sets of independent variables (e.g., demographic variables, chronic health conditions, hypertension and diabetes treatments, depressive symptoms) in order to determine which variables were associated with US – English differences in cognitive score.

All analyses used the HRS or ELSA population weights to adjust for survey non-response and for the complex sampling design (stratification and clustering) of each study.

The University of Michigan Medical School Institutional Review Board (IRB) reviewed this study and determined that it was exempt from IRB review since all data used for the study were publicly available.

## Results

### Characteristics of the US and English Study Populations

Table 1 shows the characteristics of the US and English cohorts. The US cohort included a slightly higher proportion of women. Educational attainment was significantly higher in the US, as was average household net worth (in 2002 US dollars). The English cohort reported significantly lower levels of chronic disease for every condition included in the analysis, with strikingly lower prevalence for a number of conditions. For instance, only 9% of English adults reported a diagnosis of diabetes compared to 16% of the US adults. Similarly, 45% of English adults reported hypertension, compared to 57% of US adults, while only 8% reported cancer in England compared to 18% in the US.

**Table 1:** Characteristics of the US and English cohorts, age 65+, 2002

**Variable**	**US** **(N = 8,299)**	**England** **(N = 5,276)**	P value*
Age			.07
65 – 74	51.2	54.0	
75 – 84	38.4	35.9	
≥ 85	10.4	10.2	
Mean ± SE	75.0 ± .14	74.7 ± .12	.06
Gender			<.001
Male	40.5	42.9	
Female	59.5	57.1	
Education**			<.001
Low	61.3	70.7	
Middle	18.9	10.9	
High	19.8	18.4	
Net worth (2002 US Dollars)			.01
≤ $83,000	29.0	33.5	
$83,001 – $318,000	34.9	33.1	
> $318,000	36.1	33.4	
Mean ± SE	357,700 ± 17,500	251,800 ± 6,400	<.001

Chronic Conditions			
Stroke	8.0	6.9	.03
Diabetes	16.4	9.0	<.001
Heart disease	30.9	28.6	.01
Hypertension	56.6	44.8	<.001
Lung disease	11.3	8.1	<.001
Cancer	17.6	8.0	<.001
# of ADLs† impaired			<.001
0	77.9	71.9	
1 – 3	19.2	24.4	
4 – 6	2.9	3.7	
Mean ± SE	.44 ± .02	.55 ± .02	<.001
# of IADLs‡ impaired			<.001
0	86.5	82.6	
1 – 3	12.4	16.3	
4 – 5	1.1	1.1	
Mean ± SE	.23 ± .01	.28 ± .01	.001

CES-D (Depressive symptoms)			<.001
0	43.6	36.6	
1 – 3	41.8	45.9	
4 – 8	14.6	17.5	
Mean ± SE	1.5 ± .04	1.7 ± .03	<.001
Treatment with medications:			
Hypertension	(n = 4,664)	(n = 2,368)	
	90.5	84.9	<.001
Diabetes	(n = 1,389)	(n = 480)	
No diabetes treatment	19.7	26.5	.003
Only oral medication	62.4	54.2	
Insulin +/- oral med	17.9	19.3	
Alcohol intake (days/week)			<.001
0	53.9	15.5	
< 1	15.2	33.5	
>1 to 2	13.7	24.3	
>2	17.2	26.6	
Smoking Status			<.001
Never	42.8	35.2	
Former	47.7	52.5	
Current	9.5	12.3	

Values are weighted percentages or means derived using the HRS and ELSA respondent population weights to adjust for the complex sampling design of each survey.

* P value of chi-square or t-test for a significant difference in proportion or mean between countries.

† ADLs indicates Activities of Daily Living (eating, transferring, toileting, dressing, bathing, and walking across a room).

‡ IADLs indicates Instrumental Activities of Daily Living (preparing meals, grocery shopping, making phone calls, taking medications, managing money).

**Education categories – US: Low- ≤ 12 years of school; Mid- 13 to 15 years; High- ≥ 16 years. England: Low- lower than "O-level" (typically 0 to 11 years of school); Mid- "O-level" to lower than "A-level" (typically 12 to 13 years); High- "A-level" or higher (typically > 13 years). Categories based on Banks et al 2006. [2]

Despite lower levels of chronic disease, English adults reported more limitations in ADLs and IADLs, as well as more depressive symptoms. Among those with hypertension or diabetes, US adults were significantly more likely to be taking medications for these conditions. English adults were more likely to be current smokers and drank on more days per week than those in the US.

### Cognitive Function in the US and England

**Figure 1:**
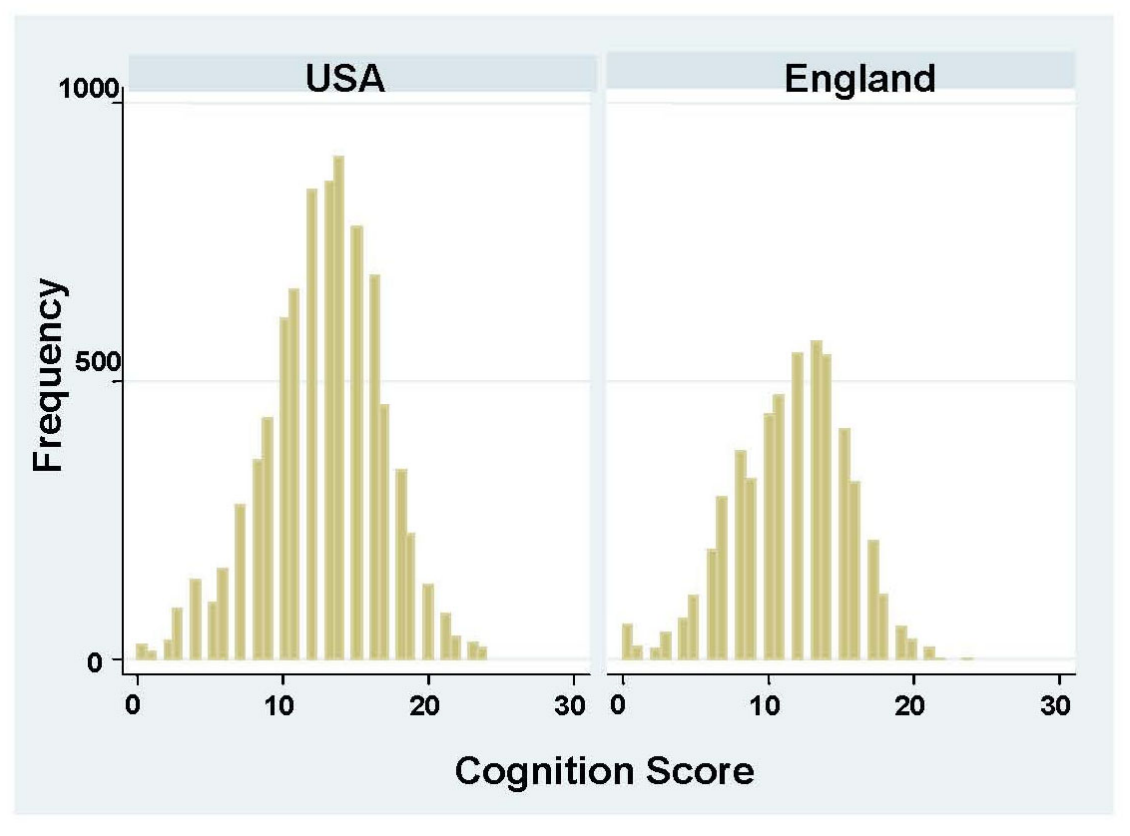
**Distribution of scores on the 24-point combined cognitive scale, by country**.

Unadjusted mean scores, as well as age- and sex-adjusted means, for each cognitive test, by country, are shown in Table 2. Overall, English adults performed significantly worse on the combined 24-point cognitive scale, scoring 1.4 points lower than US adults (a 12% relative difference, P < .001). Performance on the delayed recall test showed the greatest difference between countries, with English adults scoring 1.0 points lower than US adults on the 10-point scale (24% relative difference, P < .001). Performance on immediate recall was also significantly worse in England, while scores for orientation were similar in the two countries. Differences between the US and England for the age- and sex-adjusted means were similar to the unadjusted values. Figure 1 shows a frequency plot of the distribution of scores on the combined 24-point scale in each country.

**Table 2:** Cognitive function in the US and England, age 65+, 2002

	Unadjusted			Age- and Sex-Adjusted		
**Cognitive Test**	**US** **(N = 8,299)**	**England** **(N = 5,276)**	P Value*	**US** **(N = 8,299)**	**England** **(N = 5,276)**	P Value*

Immediate Recall (Mean ± SE)	5.1 ± .03	4.7 ± .03	<.001	5.3 ± .04	4.8 ± .04	<.001
Delayed Recall	4.1 ± .03	3.1 ± .03	<.001	4.3 ± .05	3.3 ± .05	<.001
Orientation	3.6 ± .01	3.6 ± .01	.2	3.6 ± .02	3.6 ± .02	.2
Combined Scale	12.8 ± .06	11.4 ± .06	<.001	13.2 ± .09	11.7 ± .08	<.001

* P value of t-test for a significant difference in mean between countries.

Table 3 shows cognitive performance in the US and England, stratified by age, gender, and education. As expected, cognitive performance was generally worse among older individuals in both countries, but the age-related difference in cognitive performance was more significant in England (P = .03 for Age × Country interaction). For instance, the mean score for the combined cognitive scale among English adults was 12.5 for the youngest age group (65–74) and 8.3 for the oldest age group (85+) (4.2 points, a 34% relative difference), while the mean scores in the youngest and oldest group in the US were 13.8 and 10.1, respectively (3.7 points, 27% relative difference). The largest US-English difference in total cognitive score was, therefore, in the oldest age group (1.8 point US-English difference for those 85+, compared to a 1.3 point difference for those 65–74).

**Table 3:** Cognitive function in the US and England, age 65+, by selected variables, 2002

	**Immediate Recall**		**Delayed Recall**		**Orientation**		**Combined**	
**Variable**	**US**	**England**	**US**	**England**	**US**	**England**	**US**	**England**

Age								
65 – 74	5.6	5.2	4.6	3.6	3.6	3.7	13.8	12.5
75 – 84	4.9	4.3	3.6	2.6	3.7	3.6	12.2	10.5
≥ 85	4.0	3.4	2.7	1.6	3.5	3.3	10.1	8.3

* Test (Age × Country)	.1		.9		<.001		.03	

Gender								
Male	4.9	4.6	3.8	3.0	3.6	3.6	12.2	11.2
Female	5.3	4.7	4.2	3.1	3.6	3.6	13.2	11.5

* Test (Gen. × Country)	<.001		.001		1		<.001	

Education								
Low	4.9	4.4	3.8	2.7	3.6	3.6	12.2	10.7
Middle	5.4	5.3	4.4	3.8	3.7	3.7	13.4	12.7
High	5.7	5.6	4.6	3.9	3.7	3.7	14.0	13.2

* Test (Edu. × Country)	<.001		<.001		.4		<.001	

* P value of a test for a significant interaction between the selected variable and country.

Table 4 reports the results of 8 different OLS regression models with the combined 24-point cognitive score as the dependent variable, using pooled data from the US and England. The country variable in the first row of the table represents the cognitive performance of US adults compared to English adults (reference group). Model 1 shows the unadjusted 1.43 point difference in mean score between US and English adults already noted in Table 2. Higher levels of education and net worth (Model 3) in the US accounted for some of the better cognitive performance in the US (coefficient on the country variable decreased from 1.46 to 1.30). While hypertension treatment was associated with significantly better cognitive performance (Model 5, 0.56 points) and hypertension treatment was more common in the US than England, this did not account for a large part of the US-English cognitive performance difference when holding sociodemographic and cardiovascular risks constant (country coefficient decreased from 1.35 to 1.33). The higher prevalence of depressive symptoms in England accounted for the greatest proportion of the poorer cognitive performance in England compared to the US (country coefficient decreased from 1.33 to 1.16 with the addition of controls for depressive symptoms in Model 6). Drinking alcohol was associated with better cognitive function compared to those who reported no alcohol intake. English adults were more likely to drink some alcohol, so the addition of controls for alcohol intake resulted in an increase in the US-English difference in adjusted cognitive score (country coefficient increased from 1.16 to 1.43 between Models 6 and 7). In the fully adjusted model (Model 8), being in the oldest age group was associated with the biggest decrement in cognitive function, while female gender and high levels of education were associated with the largest increment in cognitive function. Stroke and hypertension were also associated with significantly poorer cognitive function, while treatment with anti-hypertensive medication was associated with significantly better cognitive function.

**Table 4:** Cognitive function in the US and England, 2002

**Variable**	**Model 1**	**Model 2**	**Model 3**	**Model 4**
Country (England = Ref)	1.43* (1.3 – 1.6)	1.46* (1.3 – 1.6)	1.30* (1.1 – 1.4)	1.35* (1.2 – 1.5)

Age				
65 – 74		Ref.	Ref.	Ref.
75 – 84		-1.70* (-1.9 – -1.5)	-1.63* (-1.8 – -1.4)	-1.55* (-1.8 – -1.4)
≥ 85		-3.85* (-4.2 – -3.5)	-3.61* (-3.9 – -3.3)	-3.52* (-3.8 – -3.2)

Female gender		1.07* (.9 – 1.3)	1.37* (1.2 – 1.5)	1.32* (1.1 – 1.5)

Education				
Low			Ref.	Ref.
Middle			.96* (.7 – 1.2)	.94* (.7 – 1.1)
High			1.48* (1.2 – 1.7)	1.45* (1.2 – 1.7)

Net Worth (2002 $s)				
≤ 80,000			Ref.	Ref.
80,001 – 262,000			.98* (.7 – 1.2)	.91* (.6 – 1.2)
> 262,000			1.38* (1.1 – 1.6)	1.26* (1.0 – 1.5)

Cardiovascular Risks				
Stroke				-1.0* (-1.4 – -.6)
Diabetes				-.39* (-.6 – -.1)
Hypertension				-.04 (-.2 – .1)
Heart disease				-.19 (-.4 – .1)

**Variable**	**Model 5**	**Model 6**	**Model 7**	**Model 8**

Country (England = Ref)	1.33* (1.2 – 1.5)	1.16* (1.0 – 1.3)	1.43* (1.3 – 1.6)	1.43* (1.3 – 1.6)

Age				
65 – 74	Ref.	Ref.	Ref.	Ref.
75 – 84	-1.56* (-1.8 – -1.4)	-1.53* (-1.7 – -1.3)	-1.50* (-1.7 – -1.3)	-1.5* (-1.7 – -1.3)
≥ 85	-3.52* (-3.8 – -3.2)	-3.45* (-3.7 – -3.2)	-3.37* (-3.6 – -3.1)	-3.4* (-3.6 – -3.1)

Female gender	1.31* (1.1 – 1.5)	1.37* (1.2 – 1.6)	1.43* (1.2 – 1.6)	1.4* (1.2 – 1.6)

Education				
Low	Ref.	Ref.	Ref.	Ref.
Middle	.94 (.7 – 1.2)	.89 (.7 – 1.1)	.82 (.60 – 1.0)	.79 (.6 – 1.0)
High	1.45* (1.2 – 1.7)	1.39* (1.1 – 1.7)	1.31* (1.0 – 1.6)	1.3* (1.0 – 1.6)

Net Worth (2002 $s)				
≤ 82,000	Ref.	Ref.	Ref.	Ref.
82,001 – 262,000	.90 (.6 – 1.2)	.83 (.6 – 1.1)	.77 (.5 – 1.0)	.73 (.5 – 1.0)
> 262,000	1.25* (1.0 – 1.5)	1.15 (.9 – 1.4)	1.04 (.8 – 1.3)	1.0 (.8 – 1.3)

Cardiovascular Risks				
Stroke	-.99* (-1.4 – -.6)	-.96* (-1.3 – -.6)	-.91* (-1.3 – -.6)	-.89* (-1.2 – -.6)
Diabetes	-.12 (-.6 – .4)	-.11 (-.6 – .4)	-.07 (-.5 – .4)	-.01 (-.46 – .43)
Hypertension	-.55* (-.9 – -.2)	-.46* (-.8 – -.1)	-.46* (-.8 – -.1)	-.49* (-.84 – -.15)
Heart disease	-.19 (-.4 – .1)	-.13 (-.4 – .12)	-.10 (-.3 – .1)	-.10 (-.33 – .13)

Hypertension Treatment	.56* (.2 – .9)	.50* (.1 – .8)	.50* (.1 – .8)	.52* (.2 – .9)

Diabetes Treatment				
No Treatment	Ref.	Ref.	Ref.	Ref.
Only Oral Medication	-.29 (-.8 – .2)	-.28 (-.8 – .2)	-.25 (-.7 – .2)	-.25 (-.7 – .2)
Insulin +/- Oral Med	-.57 (-1.3 – .1)	-.56 (-1.2 – .1)	-.49 (-1.2 – .2)	-.50 (-1.2 – .2)

CES-D symptoms				
0		Ref.	Ref.	Ref.
1–3		-.44* (-.6 – -.3)	-.41* (-.6 – -.2)	-.42* (-.6 – -.2)
4–8		-.93* (-1.2 – -.7)	-.88* (-1.1 – -.6)	-.88* (-1.1 – -.6)

Alcohol				
0			Ref	Ref
< 1			0.74* (.5 – 1.0)	0.75* (.5 – 1.0)
>1 to 2			0.71* (.4 – 1.0)	0.72* (.4 – 1.0)
>2			0.50* (.2 – .8)	0.51* (.2 – .8)

Smoking Status				
Never				Ref.
Former				-.11 (-.3 – .1)
Current				-.20 (-.5 – .1)

Other Chronic Conditions				
Lung disease				.06 (-.2 – .3)
Cancer				.11 (.-1 – .3)

* P <.05

## Discussion

In this first international comparison of cognitive function in nationally representative samples of older adults in the United States and England, US adults performed better than their English counterparts. Using the same cognitive tests administered in the same year, US adults showed significantly better performance on standard tests of memory. The US cognitive advantage was greatest for the "oldest-old," those aged 85 and older. On a population level, the overall difference in cognitive performance between the two countries was quite large, approaching the magnitude associated with about 10 years of aging.

The better cognitive performance of US adults in our study was surprising since, as was found for younger adults aged 55–64 in a recent report, [2] we found that older US adults had a significantly higher prevalence of cardiovascular risk factors (hypertension and diabetes), heart disease, and stroke. Since increasing evidence suggests that cardiovascular risk factors and cardiovascular disease are associated with cognitive decline and poorer cognitive function, [4,17] the higher burden of these conditions among US adults would seem to predict poorer cognitive function compared to English adults with a lower burden of these conditions.

What might account for the apparently better "brain health" among older US adults compared to English adults in the face of a significantly higher burden of cardiovascular risk factors and disease? Our study suggests a number of possibilities. First, US adults had significantly higher levels of education and wealth than English adults in 2002, and these factors accounted for some of the better US cognitive performance in our multivariable analyses. More years of formal education is associated with a reduced risk of cognitive decline and dementia, [18,19] likely through multiple causal pathways, including a direct effect on brain development and function, [20] better health behaviors, [21,22] more cognitively stimulating occupations, [19,23] and a safer and more enriched social environment.[24] Greater wealth is also associated with better cognitive function, likely sharing many of the same causal pathways with education.[6,25]

Another interesting and important difference between US and English adults that may have accounted for some of the US cognitive advantage was the significantly lower level of depressive symptoms reported by US adults. Depression is associated with worse cognitive function, although this relationship is likely complex and bidirectional, with depression possibly being a risk factor for cognitive decline, and early cognitive decline possibly leading to depressive symptoms.[26] We found a significant dose-response relationship between the number of reported depressive symptoms and cognitive performance, with those reporting 4 to 8 depressive symptoms scoring nearly 1 point lower on the cognitive scale in our fully adjusted model (Model 8 in Table 4). In our multivariable models, the greater prevalence of depressive symptoms in England explained a portion of the poorer cognitive performance among older English adults. Making valid comparisons of the prevalence of diagnosed clinical depression in different countries is difficult due to differences in study populations and diagnostic criteria. A study of depression prevalence among adults in European countries found significant international variation, from about 9% in Iceland to 24% in Germany.[27] In England, depression prevalence was 10% in a sample from Liverpool and 17% in London. In the United States a large community-based study in the early 1990s found a 10% prevalence of depression among adults.[28] Interestingly, and perhaps important to the interpretation of our findings, fewer than 15% of depressed adults in these English samples were receiving medication to treat their depression, while a study of US adults during the same time period (mid-1990s), found that nearly 75% of depressed individuals were receiving medication therapy.[29] Future research should explore whether more widespread use of anti-depressant medication in the United States may be one reason for the lower level of depressive symptoms, and in turn, the better cognitive performance of older adults in the US compared to England that we found in our study.

One significant difference in health behaviours between the US and England – the consumption of alcohol – likely favoured the cognitive performance of older adults in England. We have previously reported that moderate alcohol consumption, compared to abstinence, was associated with better cognition among those aged 50 and older in the 2002 wave of the ELSA.[30] We found a similar positive relationship between alcohol consumption and cognition in those aged 65 and older, with those reporting some alcohol intake showing significantly better cognitive function than those who reported abstaining from drinking alcohol. More than 50% of US adults reported no alcohol intake compared to only 15.5% of English adults.

Finally, while US adults reported a higher prevalence of hypertension, they also were more likely to be taking medications to treat hypertension (91% vs. 85% of those with hypertension, p < .001). This finding is in line with a prior cross-national study of hypertension treatment in the 1990's showing a greater likelihood of any hypertension treatment among hypertensive US adults (age 35 to 64) compared to English adults, as well as more aggressive blood pressure lowering among those being treated (84% of treated US adults had a blood pressure of < 160/95 compared to 73% of English adults).[31] A number of observational studies have shown a relationship between hypertension and an increased risk for cognitive impairment, [3,32] as well as a protective effect of antihypertensive therapy for preventing cognitive decline.[33,34] Similarly, in our study self-reported use of antihypertensive medications was associated with significantly better cognitive function, controlling for all other covariates. However, while some randomized controlled trials of antihypertensive therapy have shown a benefit for cognitive function, [35] other RCTs have not shown a clear benefit.[36]

To test the hypothesis that more aggressive and effective hypertension treatment in the US vs. England (among those receiving treatment) might be contributing to the US cognitive advantage, we performed an additional regression analysis limited to those with hypertension. After controlling for all of the variables in our analysis (Model 8), an interaction term for hypertension treatment × country (England as reference) was positive (coefficient = 0.64 points) and statistically significant (P = .014). In this sub-analysis, hypertension treatment in the US was associated with a 0.5 point higher cognitive score (among those with hypertension, after controlling for all other covariates), while hypertension treatment in the UK was associated with a 0.1 point lower cognitive score. Future research with more detailed data on hypertension treatment (e.g., number of medications, type of medications, and dose of medications) and measured blood pressure is required to better assess whether more aggressive hypertension treatment in the US is, in fact, helping to protect cognitive function more effectively than in England.

The fact that the greatest cognitive advantage for US adults in our study was among the oldest-old may also support the hypothesis that more aggressive diagnosis and treatment of hypertension, and possibly other cardiovascular risks, in the US in middle-age and older adults leads to less significant cognitive decline among the oldest-old. Given the significant public health and cost implications of cognitive decline and the incidence of dementia in aging populations around the world, future research to identify whether more aggressive treatment of cardiovascular risks such as hypertension, hypercholesterolemia, and obesity leads to improved brain health among older adults could pay significant public health dividends.

The strengths of our study include the large nationally representative samples of adults in the US and England, and the direct assessment of cognition using the same cognitive tests administered in the same year. There are also a number of potential limitations of our study that are important to consider when interpreting our results. First, while both the HRS and ELSA are nationally representative samples, differences between the two studies in overall response rates, and in methods for the recruitment of proxy respondents to answer for sample members, could have important implications for the comparison of cognitive function of those included in our analysis. The overall response rate among all eligible respondents was 87% for the HRS in 2002 and 67% for ELSA. The HRS also included more proxy respondents compared to ELSA in 2002. Among white respondents aged 65 and older in 2002, 1,171 (12%) were represented by a proxy in the HRS, compared to only 96 (2%) in ELSA. If the difference in proxy representation between the two studies is due to the HRS being more likely to use a proxy for a respondent with impaired cognitive function (whereas the ELSA would still use a self-report interview), this could lead to the pattern of better apparent cognitive performance among the HRS self-respondents compared to the ELSA self-respondents included in our study. To assess this possibility, we compared the characteristics of respondents represented by a proxy in the HRS and ELSA in 2002, and did not find evidence that HRS respondents represented by a proxy were "sicker" or more likely to have impaired cognition than those in the ELSA. For instance, compared to ELSA proxies, HRS proxies represented individuals who were younger (81% were aged < 85 in HRS compared to 61% in ELSA, P < .01) and had higher net worth (31% in the top tertile in the HRS, compared to 24% in ELSA, P < .05). In addition, proxies in the HRS rated the overall cognitive function of those whom they represented as somewhat better than ELSA proxies using the same informant scale (the Informant Questionnaire for Cognitive Decline in the Elderly.[37] Taken together, these comparisons of respondents represented by a proxy in the two studies suggest that the poorer cognitive performance of English adults in our study is not an artifact of differences in the utilization of proxy respondents.

While the same cognitive tests were administered in both the HRS and ELSA, one important difference in the administration of the test should be considered when interpreting the results. About 70% of the HRS sample was interviewed by telephone and 30% in-person, while all ELSA interviews were in-person. If telephone administration of the cognitive tests is associated with systematically better performance compared to in-person administration, this could explain some of the HRS cognitive advantage that we found. However, two prior studies have examined the impact of telephone vs. face-to-face administration of the HRS cognitive tests, and found no significant differences in test scores for the different survey modes.[38,39] Finally, it should be noted that our OLS regression analysis may not have accurately identified non-linear relationships between the predictor variables and the cognitive score outcome.

## Conclusion

In conclusion, we found that despite a higher prevalence of cardiovascular risks and cardiovascular disease among older US adults, they performed significantly better than their English counterparts on tests of memory, suggesting an advantage in cognitive health in the United States. While we were unable to confidently identify the cause or causes of this US advantage, higher levels of education and wealth, lower levels of depressive symptoms, and more aggressive treatment of cardiovascular risks such as hypertension, may be important contributing factors. Given the growing number of older adults worldwide, future cross-national studies aimed at identifying the medical and social factors that might prevent or delay cognitive decline in older adults would make important and valuable contributions to public health.

## Competing interests

The authors declare that they have no competing interests.

## Authors' contributions

KL conceived and participated in the design of the study, helped with statistical analysis, analyzed and interpreted data, and drafted the manuscript. DL conceived and participated in the design of the study, helped acquire the data, helped with statistical analysis, and analyzed and interpreted data. IL conceived and participated in the design of the study, and analyzed and interpreted data. DW conceived and participated in the design of the study, and analyzed and interpreted data. RW analyzed and interpreted data. MK helped acquire the data, led the statistical analysis, and analyzed and interpreted data. FH conceived and participated in the design of the study, and analyzed and interpreted data. All authors provided critical revision of the manuscript, and read and approved the final manuscript.

## Pre-publication history

The pre-publication history for this paper can be accessed here:



## References

[B1] Cigolle CT, Langa KM, Kabeto MU, Tian Z, Blaum CS (2007). Geriatric conditions and disability: the Health and Retirement Study. Ann Intern Med.

[B2] Banks J, Marmot M, Oldfield Z, Smith JP (2006). Disease and disadvantage in the United States and in England. JAMA.

[B3] Kivipelto M, Helkala EL, Laakso MP (2001). Midlife vascular risk factors and Alzheimer's disease in later life: longitudinal, population based study. BMJ.

[B4] Langa KM, Foster NL, Larson EB (2004). Mixed dementia: emerging concepts and therapeutic implications. JAMA.

[B5] Kumari M, Marmot M (2005). Diabetes and cognitive function in a middle-aged cohort: findings from the Whitehall II study. Neurology.

[B6] Langa KM, Larson EB, Karlawish JH (2008). Trends in the prevalence and mortality of cognitive impairment in the United States: is there evidence of a compression of cognitive morbidity?. Alzheimers Dement.

[B7] Kaiser Family Foundation Health Care Spending in the United States and OECD Countries. http://www.kff.org/insurance/snapshot/chcm010307oth.cfm.

[B8] Juster FT, Suzman R (1995). An overview of the Health and Retirement Study. Journal of Human Resources.

[B9] Marmot M, Banks J, Blundell R, Lessof C, Nazroo J (2003). Health, wealth, and lifestyles of the older population in England: The 2002 English Longitudinal Study of Ageing.

[B10] Health and Retirement Study. http://hrsonline.isr.umich.edu.

[B11] English Longitudinal Study of Ageing. http://www.ifs.org.uk/elsa.

[B12] Ofstedal MB, Fisher G, Herzog AR Documentation of Cognitive Functioning Measures in the Health and Retirement Study. http://hrsonline.isr.umich.edu/sitedocs/userg/dr-006.pdf.

[B13] Steffick D Documentation of Affective Functioning Measures in the Health and Retirement Study. http://hrsonline.isr.umich.edu/sitedocs/userg/dr-005.pdf.

[B14] Turvey CL, Wallace RB, Herzog R (1999). A revised CES-D measure of depressive symptoms and a DSM-based measure of major depressive episodes in the elderly. Int Psychogeriatr.

[B15] Fonda S, Herzog AR Documentation of Physical Functioning Measured in the Heath and Retirement Study and the Asset and Health Dynamics among the Oldest Old Study. http://hrsonline.isr.umich.edu/sitedocs/userg/dr-008.pdf.

[B16] FX History^®^: historical currency exchange rates. http://www.oanda.com/convert/fxhistory.

[B17] Llewellyn DJ, Lang IA, Xie J, Huppert FA, Melzer D, Langa KM (2008). Framingham Stroke Risk Profile and poor cognitive function: a population-based study. BMC Neurol.

[B18] Valenzuela MJ, Sachdev P (2006). Brain reserve and dementia: a systematic review. Psychol Med.

[B19] Stern Y, Gurland B, Tatemichi TK, Tang MX, Wilder D, Mayeux R (1994). Influence of education and occupation on the incidence of Alzheimer's disease. JAMA.

[B20] Stern Y (2006). Cognitive reserve and Alzheimer disease. Alzheimer Dis Assoc Disord.

[B21] Gatz M, Prescott CA, Pedersen NL (2006). Lifestyle risk and delaying factors. Alzheimer Dis Assoc Disord.

[B22] Carlson MC, Helms MJ, Steffens DC, Burke JR, Potter GG, Plassman BL (2008). Midlife activity predicts risk of dementia in older male twin pairs. Alzheimers Dement.

[B23] Potter GG, Helms MJ, Plassman BL (2008). Associations of job demands and intelligence with cognitive performance among men in late life. Neurology.

[B24] Lang IA, Llewellyn DJ, Langa KM, Wallace RB, Huppert FA, Melzer D (2008). Neighborhood deprivation, individual socioeconomic status, and cognitive function in older people: analyses from the English Longitudinal Study of Ageing. J Am Geriatr Soc.

[B25] Cagney KA, Lauderdale DS (2002). Education, wealth, and cognitive function in later life. J Gerontol B Psychol Sci Soc Sci.

[B26] Steffens DC, Otey E, Alexopoulos GS (2006). Perspectives on depression, mild cognitive impairment, and cognitive decline. Arch Gen Psychiatry.

[B27] Copeland JR, Beekman AT, Braam AW (2004). Depression among older people in Europe: the EURODEP studies. World Psychiatry.

[B28] Kessler RC, McGonagle KA, Zhao S (1994). Lifetime and 12-month prevalence of DSM-III-R psychiatric disorders in the United States. Results from the National Comorbidity Survey. Arch Gen Psychiatry.

[B29] Olfson M, Marcus SC, Druss B, Elinson L, Tanielian T, Pincus HA (2002). National trends in the outpatient treatment of depression. JAMA.

[B30] Lang I, Wallace RB, Huppert FA, Melzer D (2007). Moderate alcohol consumption in older adults is associated with better cognition and well-being than abstinence. Age Ageing.

[B31] Wolf-Maier K, Cooper RS, Kramer H (2004). Hypertension treatment and control in five European countries, Canada, and the United States. Hypertension.

[B32] Glynn RJ, Beckett LA, Hebert LE, Morris MC, Scherr PA, Evans DA (1999). Current and remote blood pressure and cognitive decline. JAMA.

[B33] Murray M, Lane K, Gao S (2002). Preservation of cognitive function with antihypertensive medications. Archives of Internal Medicine.

[B34] Khachaturian AS, Zandi PP, Lyketsos CG (2006). Antihypertensive medication use and incident Alzheimer disease: the Cache County Study. Arch Neurol.

[B35] Forette F, Seux ML, Staessen JA (1998). Prevention of dementia in randomised double-blind placebo-controlled Systolic Hypertension in Europe (Syst-Eur) trial. Lancet.

[B36] Qiu C, Winblad B, Fratiglioni L (2005). The age-dependent relation of blood pressure to cognitive function and dementia. Lancet Neurol.

[B37] Jorm AF (1994). A short form of the informant questionnaire on cognitive decline in the elderly (IQCODE): development and cross-validation [published erratum appears in Psychol Med 1995 Mar;25(2):437]. Psychological Medicine.

[B38] Herzog AR, Wallace RB (1997). Measures of cognitive functioning in the AHEAD Study. J Gerontol B Psychol Sci Soc Sci.

[B39] McArdle JJ, Fisher GG, Kadlec KM (2007). Latent variable analyses of age trends of cognition in the Health and Retirement Study, 1992–2004. Psychol Aging.

